# IC-Based Rectification Circuit Techniques for Biomedical Energy-Harvesting Applications

**DOI:** 10.3390/mi13030411

**Published:** 2022-03-05

**Authors:** Cihun-Siyong Alex Gong

**Affiliations:** 1 Department of Electrical Engineering, School of Electrical and Computer Engineering, College of Engineering, Chang Gung University, Taoyuan 33302, Taiwan; alexgong@mail.cgu.edu.tw; 2 Portable Energy System Group, Green Technology Research Center, College of Engineering, Chang Gung University, Taoyuan 33302, Taiwan; 3 Department of Ophthalmology, Chang Gung Memorial Hospital, Linkou, Taoyuan 33305, Taiwan

**Keywords:** biomedical, implantable, prostheses, biomedical, AC-DC, energy harvesting, conversion efficiency, rectifier

## Abstract

Energy harvesting can be achieved through many different mechanisms. Such technology has been drawing researchers’ attention to its practical applications for a decade, as it can be widely applied to countless scenarios. It steals the show in the modern development of the biomedical electronics, especially implantable applications, as it allows the patients to move freely without restriction. To prolong lifetime of the battery inside/outside a patient’s body, the electrical conversion efficiency of the electronic implant is of primary importance in energy harvesting. The conversion can be achieved by a so-called miniaturized rectification circuit (also known as “rectifier”). This study aims to compare different state-of-the-art techniques focusing on the conversion efficiency of the rectification. Particular emphasis is put on semiconductor-based circuits capable of being integrated with tiny chips on the implants.

## 1. Introduction

Current IC design and foundry processes increasingly allow for the manufacture of sophisticated system-on-chip (SoC) applications, allowing for the miniaturization of integrated biomedical devices. Implantable medical chips (also referred to as “implantable microelectronics”) are cross-disciplinary integrated systems which have advanced rapidly over the past half year, producing a string of new applications for the treatment of many major neurological and physiological conditions related to visual, auditory, neuromuscular, neurological or other functions, thus offering the possibility of tremendous improvements to quality of life.

Looking ahead, health-related technologies have emerged as the focus for the next generation of industrial development, and are sure to have a far-reaching impact on human health and quality of life. To promote the development of such technologies, countries around the world are actively establishing cross-disciplinary medical research groups. Currently, applications using implantable chips allow surgeons to diagnose and formulate treatment options for brain lesions in neuropathy patients [1], while high-performance implanted chips are being developed for use in artificial ear [2] and eye [3] applications.

**Figure 1:**
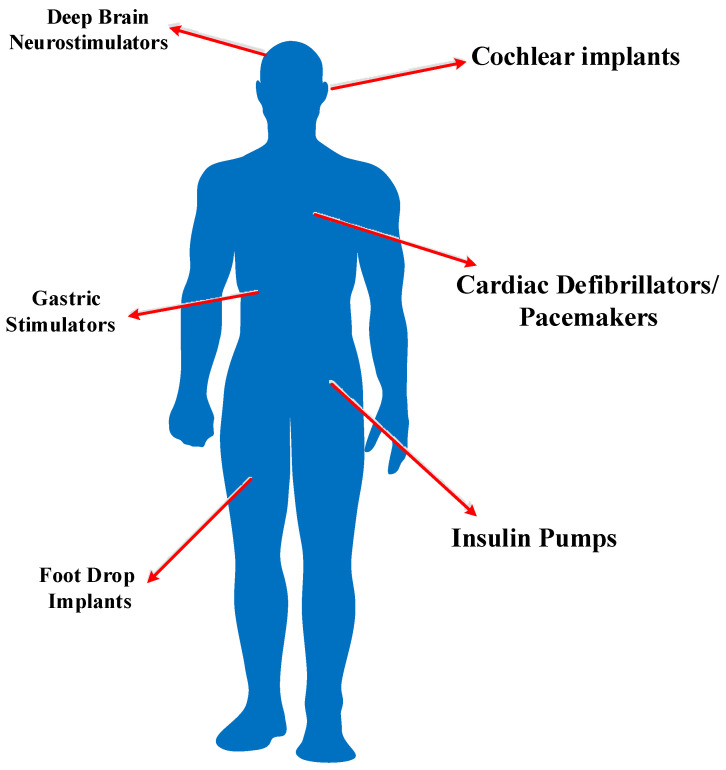
Implantable medical devices for various applications.

Figure 1 shows the implantable applications. Pacemakers are used to assist defective hearts and correct arrhythmia. When the heart beats irregularly, the pacemaker emits electrical pulses to restore the correct rhythm. Artificial ears are used to restore auditory sensitivity to patients suffering from partial or total hearing loss, with external radio components sending electronic signals and electrical power through a wireless coupling to the implanted components which reproduce external sounds in the inner ear, thus restoring hearing function. Retinal electronic implants are used to restore visual function. Unfortunately, current battery technology has not yet been able to support the permanent operation of them, and depleted batteries used in the implanted devices must be replaced.

### Wireless Implantable Medical Systems

**Figure 2:**
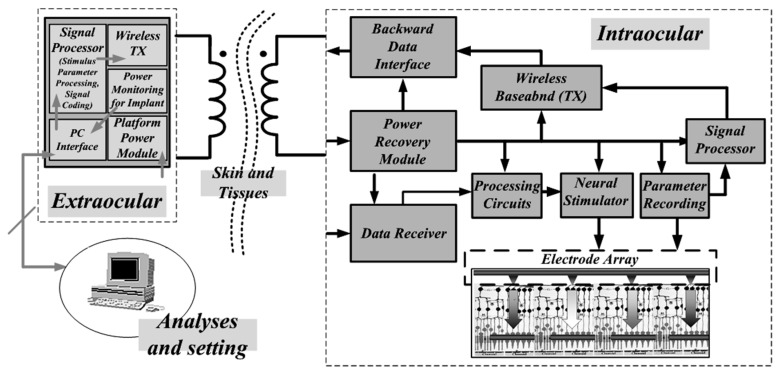
Wireless implantable system architecture for a Retinal Electronic Prosthesis with an external PC used to analyze various physiological and environmental parameters taken from intraocular sensor records.

Radio power transmission technology has been proposed to address the lifetime problem. Taking the Retinal Electronic Prostheses (REPs) as an example, the system in Figure 2 consists of a sensor interface external to the eyeball, used to capture images and other signals (not on the PC shown in the image). All captured signals are processed using parameters determined by the clinician. These parameters determine the pulse duration, stimulation time and output duration (i.e., the width of the current pulse) when the module is engaged. A series of signal processing steps follows, including coding and modulation to obtain information required for generating visual perception. This information will determine the size of the injected current generated by the microstimulator module for electrical neuronal stimulation. Finally, the integrated signal is amplified by the external platform and transmitted in vivo.

The electrodes made by MEMS technology are closely connected to the electrical stimulator and the neural signal recording circuit, and the process technology and material used will greatly affect the electrical driving ability and the clarity of the signal recording. Different electrode sizes and electrode materials will directly affect the electrode impedance, noise, and charge tolerance. Depending on the application and stimulation waveform, adjustment of electrode size and electrode spacing can significantly affect the physiological response. In addition, MEMS packaging also determines long-term success or failure of the chip.

Implantable systems use inductive coupling for reception through the built-in antenna module for the sake of chronic implantation, which consists of two parts. The first receives the carrier signal through alternating AC-DC and DC-DC modules to provide a stable power supply for the active circuit. At the same time, the processed data from the external platform are transmitted to the in vivo system for demodulation by a component which simultaneously generates a time signal for the in vivo system. Compared to approaches which supply power through a wire connected directly to the eye or that positions batteries within the eye, this power supply approach allows for perpetual use following a single operation to implant the “System on a Chip, SOC”, while also eliminating the risk of battery leakage or infection [4].

The stimulator activates at the appropriate time based on the implant demodulation and subsequent data processing. Then, the signal passes through the pre-amplifier to record the neural response, which is produced when the stimulus current reaches the activation threshold. The recorded neural response can be modulated by the on-implant modulator for external transmission. The modulating module also allows the in vitro platform to receive the recorded neural signal for analysis by the external platform (shown here as a PC) to assess implant performance and study physiological signals through the SOC. This paper primarily focuses on the AC-DC module.

## 2. Underlying Principles of Wireless Powering

### 2.1. Theory

Wireless energy transfer mainly consists of two antennas with an electromagnetic field (EM) transferring LC resonance energy between panel A and panel B. Wireless energy transmission can be basically classified as Near-Field Communication (NFC) and Far-Field Communication (FFC), where NFC is further divided into the Non-Radiative (Reactive) and Radiative (Fresnel). The key difference between these are the distance and linear dimension of the antenna. In Far Field, the electromagnetic waves are completely free from transmitter interference. The energy is transmitted over a long distance by electromagnetic waves, and can be described by the Friss Formula. Near Field can be divided into Reactive and Fresnel types. Reactive Near Field requires a much larger component than Fresnel, meaning any change in the electrical characteristics of the electrical field antenna or the magnetic characteristics of the magnetic field will have a significant impact on the impedance of the antenna input point. Fresnel Near Field is dominated by the Fresnel field, and the surrounding medium has little impact on antenna impedance. However, the distance between this area and the antenna is very small, so the antenna size is a factor that must be considered, thus the angular distribution of the Fresnel pattern is related to the distance. Overall, the characteristics of Near Field are that it has a short electromagnetic wave transmission range, but with a large energy volume and relatively small path loss. When setting conditions to limit a specific absorption rate, it can provide smaller loss than Far Field. For transmitting energy and information, these characteristics show that Far Field provides safer battery use conditions, and thus is typically used for wireless energy transmission for implanted biomedical devices.

### 2.2. Frequency Selection

From this discussion, we see that most NFC and FFC solutions use NFC for transmission in biomedical applications. Whether for RFID wireless communication of wireless charging in biomedical applications, there are strict specifications for carrier frequency and frequency bands, mainly to prevent mutual interference between wireless products. Therefore, it is necessary to formulate specifications to ensure that the frequencies used by various products will not cause interference. Most restricted frequency bands are not open, and the vast majority are restricted to commercial or military use. For example, commercial use frequencies are sold by auction to commercial bidders, while military use frequencies are allocated by governments based on national defense considerations. Some limited frequency bands are open to unrestricted public use under certain conditions, such as those developed by the International Telecommunications Union Radiocommunications Sector (ITU-R), or the Industrial, Scientific and Medical band (ISM) which is a frequency band limited for use by industrial, scientific and medical institutions.

Use of these frequencies does not require specific licensing or fees, but usage applications must comply with certain transmission power limits (generally under 1 W) and must avoid causing interference with other frequencies. For example, 2.45 GHz is the high-frequency oscillation band commonly used in microwave ovens, wireless network systems (e.g., mobile phone networks, Bluetooth, satellite TV and wireless local area networks, etc.), and sensor systems. In addition, 13.56 MHz is currently the most widely used frequency band for industry, science and medicine, thus most wireless power modules are designed to use this band, but other frequency bands must also be used depending on biomedicine application specifications. In principle, the higher the frequency band, the smaller the distance attenuation. Medical applications should not exceed 20 MHZ because frequencies above this range can be absorbed by human cells and have potentially negative health effects.

### 2.3. Wireless Powering in Medicine

Most biomedical systems transmit in a range of approximately 100 kHz to 20 MHz. Wireless energy transmission in this frequency band not only meets the requirement for wireless charging, but also provides telemetry recursive data transmission, but this is limited to the ISM Band in Table 1 Most current commercial wireless transmission specifications are still based on 6.78 MHz and 13.56 MHz, further promoted by mutual agreement between the WPC, A4WP and PMA (Table 1), thus the following sections focus on these two frequency bands and higher.

Electromagnetic induction refers to a conductor in a changing magnetic flux that generates an induced voltage called an induced voltage. If this conductor is closed into a loop, the voltage drives a flow of electrons, forming an induced current. The advantage of this approach is its low cost, while the disadvantage is that the magnetic material is not bound, and the magnetic flux of the transmitting coil will not pass through the receiving coil, thus it is necessary to closely align the wireless charger and the charging object. At present, the development of inductive wireless power transmission technology is relatively mature, with very good transmission efficiency over short transmission distances. However, as the transmission distance increases, the transmission efficiency falls very quickly, and the relative position of the coil is the greatest challenge to the development of effective applications.

Electromagnetic resonance refers to the use of resonant devices (inductors and capacitors) to cause the transmitter and receiver to reach a specific frequency which, in turn, generates magnetic resonance and thus energy. The advantages are high transmission efficiency and power. The disadvantage is the relatively high cost. High resonance frequency will impact transmission efficiency, but it is relatively easy to design applications for charging multiple devices simultaneously. At present, electromagnetic resonance technologies offer high efficiency, long transmission distance and strong tolerance to coil deviation.

Furthermore, 6.78 MHz is the standard frequency for magnetic resonance technologies, and is thus used for magnetic resonance in many cases. As previously mentioned, this technology has high transmission efficiency and energy, so 6.78 MHz wireless power transfer mostly transmits higher wattages and therefore most IC-based rectifier designs tend to use the CMOS BCD process or other processes that withstand higher voltages. The larger area brings a commensurate increase in current resistance, giving full play to the advantages of magnetic resonance technology. Commercialized designs can reach up to 1000 W, thus another type uses Discrete Components instead of IC Rectifiers. Although this requires a larger size, it can meet requirements for voltage and current resistance, usually because it can achieve high wattage charging energy, and most products that require such high energy levels provide the necessary volume. Thus, although the IC circuits in Discrete Components are relatively large, this approach meets the requirements of applications such as recharging of electric vehicles.

In addition, 13.56 MHz is used for magnetic induction technologies, and is thus used here for all magnetic induction technologies which are nearly all RFID applications. RFID offers advantages including low cost and the use of NFC (near-range wireless transmission) for wireless data transmission and charging over distances less than 1 m. However, transmission power achieved using this approach is less than that of magnetic resonance, so it is mainly used for instantaneous charging and data transfer. Recently, the active development of biomedical circuits has raised the need for data transfer with low power consumption and high durability. These requirements generally overlap with RFID, and many papers on wireless energy harvesting have used 13.56 MHz. Because of the above-mentioned low transmission power, it is suitable for use in IC-based circuits, allowing for a reduction in size and current without using high voltage resistance or requiring size parameters to withstand larger currents.

Finally, the circuit design for frequency bands exceeding 433 MHz is discussed. Compared to low frequencies, the main challenges encountered in high-frequency circuits are additional parasitic effects and matching problems. In low-frequency circuits, most of the parasitic capacitance and inductance can be regarded as infinite impedance and infinitesimal impedance. In short, most of the characteristics of low-frequency circuits serve to maintain the characteristics of transistors and circuits, and do not entail excessive parasitic effects to affect circuit performance. However, in the high-frequency band, the original parasitic capacitance and parasitic inductance are caused by the high-frequency effect, resulting in a prominent parasitic effect. A common problem is the matching of the input impedance at the two ends of the differential input. For the design of a differential pair, if the input at both ends is excessively severe because of the mismatch effect, the input voltage at both ends will not be a differential input, causing problems for the back-end circuit operation. Therefore, the designer must conduct more accurate simulations and calculations of the inductance and capacitance of the input end.

## 3. AC-DC Conversion Circuit Architecture

In Figure 2, we have seen where the Power Recovery Module is located. It serves as an AC-DC conversion system for the implanted chips. The rectifier inside is used to receive the wireless carrier transmitted through the skin. It belongs to AC-DC and plays a core role. Its corresponding input terminal is the receiving antenna inside the body (usually close to the skin). Through the concept of resonance, the induced electromotive force is generated and then induced in the body. The induced current at the receiving end forms the voltage on the receiving chip through the load, and supplies power to the implanted SOC in the body. However, because the power supply is essentially an AC signal, it needs to be rectified by a rectifier circuit, and the output waveform is an imperfect DC signal needing to be further filtered by a low-pass filter for high-frequency removal. In order to ensure that the power supply of the SOC in the body can remain stable even under the condition of receiving voltage fluctuations, it is necessary to have it passed a low-pass filter.

The downstream voltage regulator circuit is used to regulate the output of the filter, so that the power supply to the implanted SOC in the body can reach a sufficiently low ripple to ensure that the SOC in the body will not malfunction and cause physiological damage. The received voltage fluctuation is mainly due to the distance variation between the transmitter and the receiver. Owing to the nature of the system setups in hardware, their relative distance cannot be kept absolutely fixed, resulting in a change in the coupling coefficient of the two coils. Poor coupling coefficient compromises the conversion efficiency due to the increase in the distance between the transmitter in vitro and the receiver in the body.

The efficiency decayed exponentially, aggravating the difficulty of the internal circuit design of the AC-DC in the body. The rectifier itself is responsible for different forms of energy conversion, and its important performance index is the conversion efficiency, which is mainly divided into voltage conversion efficiency VCE and power conversion efficiency PCE. VCE is defined as ratio of the output voltage (numerator) to input voltage (denominator) of the rectifier circuit (expressed as percentage), while PCE is defined as the ratio of the output power (numerator) to the input power (denominator) of the rectifier circuit (also expressed as a percentage). The higher the value of the two, the better the designed rectifier. Efficiency usually comes with the trade-off of the design complexity.

### 3.1. Full-Wave Diode Bridge Rectifier

**Figure 3:**
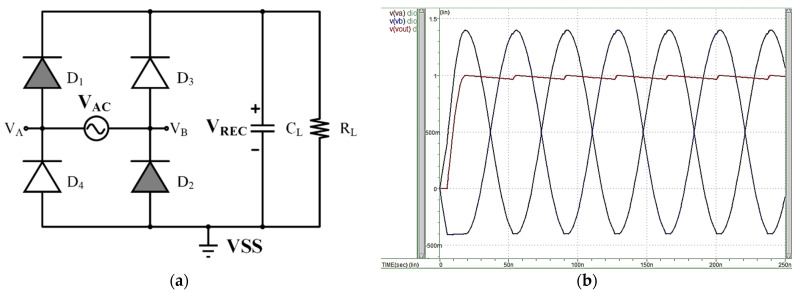
(**a**) Full-wave diode bridge rectifier architecture. (**b**) Simulated full-wave diode bridge rectifier (V_th_ ≈ 0.4 V).

Conventional rectifiers use a PN interface for a Full-wave Bridge Rectifier configured using four PN diodes or a Schlocky Barrier Diode to change the current flow. Because the diodes’ reverse bias characteristic, there is no leakage current, thus minimizing unnecessary energy loss, but the output voltage will be depleted by the diode cross-pressure (V_th_). The relatively low voltage conversion rate results in a low overall conversion rate. Figure 3a shows the circuit, while Figure 3b shows a Full-wave Bridge Rectifier simulated in a 180 nm standard CMOS single-poly and six-metal process, including 3.3-V tolerable I/O devices. The circuit design is relatively simple and stable. However, this rectifier consumes twice the diode’s threshold voltage (V_th_), with the maximum output voltage calculated by Equation (1):

(1) $$V_{out,MAX}=V_{in}-2V_{th}$$

where the diode forward bias voltage V_th_ is 700 mV, and the Schottky diode has a threshold voltage of about 300 mV. Therefore, applying the diode bridge rectifier causes an excessively large voltage drop in low-voltage implanted devices, resulting in significant reductions in voltage conversion efficiency (VCE) and power conversion efficiency (PCE). These diodes thus cannot be used in the integrated circuit, thus increasing the implant size. As chip design and manufacturing capabilities improve, the resulting implant device circuits will require less power to operate. Using a diode bridge rectifier for low-power requirements will increase the ratio of wasted voltage, resulting in the PCE and VCE having a greater influence.

### 3.2. Dual Cross-Coupled Rectifier

**Figure 4:**
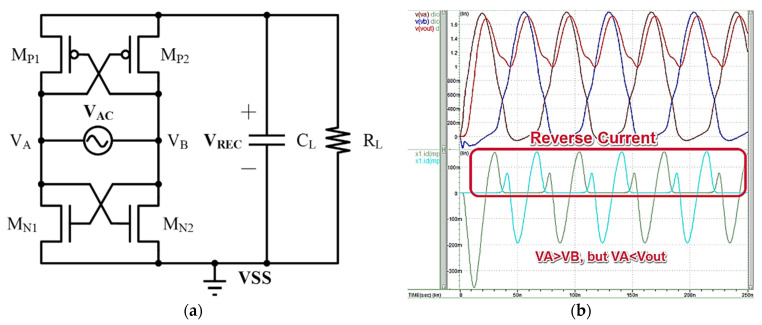
(**a**) Dual Cross-Coupled Rectifier Architecture. (**b**) Simulated Dual Cross-Coupled Rectifier (V_th_ ≈ 0.4 V).

Figure 4a shows the above-mentioned Dual Cross-Coupled Rectifier, which presents an advantage in that it uses negative-resistance architecture to load the pull-up and pull-down paths, thus completely eliminating the pressure drop caused by the rectifier on the diode. The negative resistance cross-coupled circuit acts as a switch which is open if only a very small channel resistance is selected, thus greatly reducing the voltage drop. The circuit operates as follows: When the V_A_ is high and V_B_ is low, M_N2_ and M_P1_ are on. At this time, V_B_ will connect to the ground, and V_A_ will charge the capacitor. Otherwise, when V_B_ is high and V_A_ is low, M_N1_ and M_P2_ are open, V_A_ is grounded and V_B_ charges the capacitor. However, after V_B_ reaches its peak voltage, V_B_ is still higher than V_A_ despite the falling input voltage. At this time, the switch is not closed, and the charging path is still present. At this time, if V_B_ is lower than V_REC_, this path becomes the path of discharge. Therefore, the Dual Cross-Coupled Rectifier will continue to charge and discharge the capacitor. The Dual Cross-Coupled Rectifier overcomes the dual diode voltage drop of the bridge rectifier, thus improving power conversion efficiency. However, the constant charging and discharging of the reverse current will result in significant power consumption by the rectifier. Figure 4b shows a simulation of the Dual Cross-Coupled Rectifier, indicating that it can overcome the diode’s voltage drop, but will produce a reverse current.

### 3.3. Hybrid Rectifier

**Figure 5:**
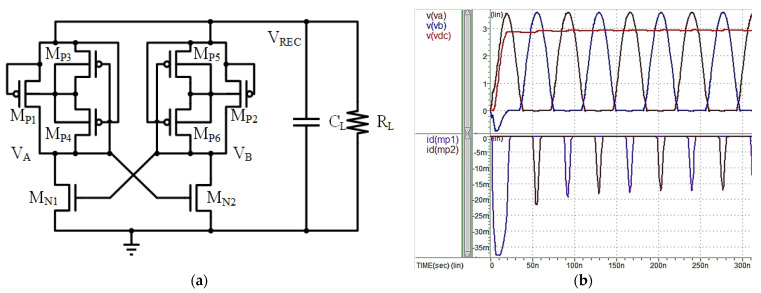
(**a**) Hybrid Rectifier Architecture. (**b**) Simulated Hybrid Rectifier.

In addition to the above-mentioned simple structures, a hybrid rectifier, as shown in Figure 5a, provides a set of diodes to prevent current flow [5]. The conduction path only requires a single diode to prevent a countercurrent, thus M_P1_ and M_P2_ can, respectively, be used as the positive and negative half-cycle anti-countercurrent diodes. Once the circuit achieves anti-countercurrent function, the lower half exhibits essentially no drop in voltage consumption in the negative resistance cross-coupled circuit (i.e., M_N1_ and M_N2_). To effectively prevent the Latch-up effect (parasitic vertical PNP transistors), and to reduce substrate leakage, the PMOS body must be kept at the highest level, thus requiring auxiliary transistors M_P3_~M_P6_. In terms of left/right symmetry, we take the right half as an example. When the node V_A_ rises to the high level, M_N1_ will first use VSS to connect to VB. Next, M_P1_ will turn on to form a loop to cause capacitance charging between V_REC _ and VSS. Because the gate and transistor source for M_P1_ and M_P3_ are linked together, when M_P1_ is turned on, M_P3_ will also turn on. The V_A_ potential will be received by the M_P3_ drain/body, causing the N-Well to remain close to the maximum potential (i.e., the maximum potential minus a P-N Junction conduction voltage). When the V_A_ voltage drops below V_REC _ + |Vtp| hours (where |Vtp| is the absolute value of the PMOS threshold voltage, which here is M_P1_), then M_P1_ and M_P3_ will both be OFF. When V_A_ falls |Vtp| below V_REC_, M_P4_ will be on, and the body will receive V_REC _ to ensure the body is at the highest potential, i.e., that of the source. The hybrid architecture combines the advantages of the bridge rectifier and the cross-coupled rectifier, incurring a smaller diode voltage drop than the bridge rectifier, thus improving power conversion efficiency. It also incurs less countercurrent than the cross-coupled rectifier, resulting in further power savings. Simulation results are shown in Figure 5b.

### 3.4. CMOS Active Rectifier Using Pull-Up V_th_ Elimination Technology

**Figure 6:**
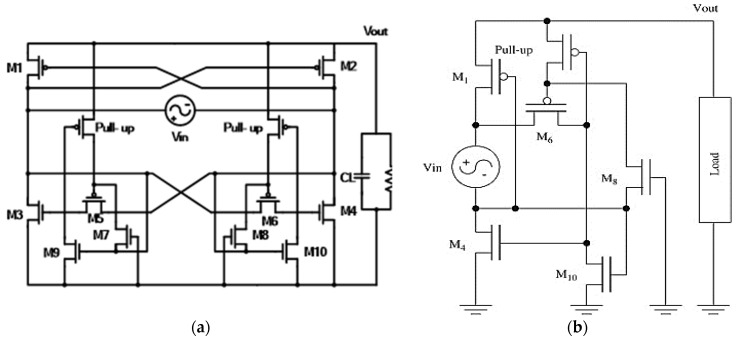

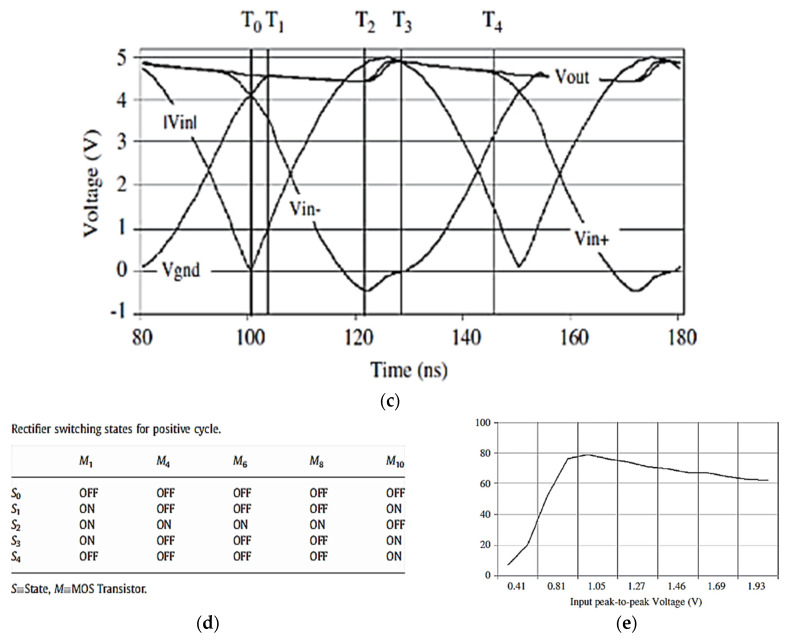
(**a**) Active rectifier using pull-up technology [6]. (**b**) Schematic diagram of the half-wave cycle for each transistor initialization [6]. (**c**) Simulated pull-up active rectifier output results [6]. (**d**) Rectifier switching states for positive cycle [6]. (**e**) power conversion efficiency and input Voltage scan [6]. Reprinted with permission from Ref. [6]. Copyright © 2009 Elsevier Ltd.

Active rectifiers using pull-up technology [6] use the M_1_ to M_4_ modules to construct key rectified transistors with upstream M_1,2_ using PMOS, and downstream M_3,4_ using NMOS. When V_in_ > V_out_, it provides a current path from the input to the load terminals and uses the pull-up control circuit to integrate M_5_–M_8_ for the M_9,10_ control gate voltage, with the circuit shown in Figure 6a. M_9,10_ causes the M_3,4_ gate voltage to ground. When V_in_ < V_out_, the control M_3,4_ is in the cutoff area, and M_1,2_ and M_3,4_ simultaneously turn off the path from the charged load capacitor’s reflow current to the input terminal.

During the positive period, M_1_ turns on and then activates the connection between the output and input terminals such that the signal source which ignores the possible resistance drop is set to the grounded voltage relative to V_out_-V_in_. If the input terminal pressure V_in_ > V_out_, the input terminal voltage is lower than the ground. Thus, when the input voltage is lower than the ground for one V_th_, M_7,8_ is turned on, conducting low voltage to the M_5,6_ gate. This forces M_5,6_ to open, and also pull up M_4_ to the open state. Figure 6b shows the partial circuit of the positive half-wave cycle. The simulated pull-up for the active rectifier output results are shown in Figure 6c, while the state of each transistor in each half-wave cycle is shown in Figure 6d. When V_in_ < V_out_, M_7,8_ is off, and the pull-up PMOS transistor is used to connect the M_5,6_ gate to the V_out_, and the M_3,4_ transistor gate is driven by the same voltage. In the negative half-cycle, the same method is used, and these two rectifier circuits are used to achieve rectification. A frequency of 10 MHz and an input voltage Vp-p of 2.2 V achieves the best PCE of 79%, and the relationship between measured power conversion efficiency and input voltage scan is shown in Figure 6e.

### 3.5. CMOS Active Rectifier with Resistance Division V_th_ Elimination Technology

This rectifier uses a threshold voltage cancellation technique to reduce the voltage drop at the conduction threshold [7], using two PMOSs for the M_5_ and M_6_ upstream of the rectifier, and using a cross-coupled bias to reduce M_5_ and M_6_ conduction when the threshold voltage is affected. The other part uses two NMOSs as upstream rectifiers for M_3_ and M_4_, but the M_3_ and M_4_ transistor gate bias voltage is connected with the bias obtained by the M_1_ and M_2_ transistors. This dynamic bias voltage is lower than the threshold voltage of M_3_ and M_4_ and its average voltage can be adjusted according to the size of M_1_ and M_2_ along with the resistance values R_1_ and R_2_. When one of the M_3_ or M_4_ transistors is turned on and the other is off, the high bias voltage will be less than R_1_ and R_2_ during the turn-on period. However, the actual bias voltage is too close to the transistor’s threshold voltage, causing current leakage from the output terminal’s C_L_ to the input terminal. Therefore, completely eliminating the threshold voltage bias value will reduce the PCE. Thus, 70% of the M_3_ and M_4_ are conductively and non-conductively connected to the cross-coupled resistor to store the voltage to achieve a better tolerance range between 65% and 75%. The transistors M_3_ and M_4_ conduct when the negative RF signal is 150 mV lower than V_REC_. The simulated analog waveform where, following distribution, the R_L_ for the rectifier is 10 kΩ and C_L_ = 1 nF. This technology achieves a PCE of 85.8% at a frequency of 40.69 MHz and a load resistance of 8 KΩ.

### 3.6. Bootstrapped Rectifier

A bootstrapped rectifier circuit is presented in [8]. The cross-coupled MOS structure is still used as negative resistance, operating like a switch without bipolar pressure drop issues, while the upper half is used like a belt circuit. Because the NMOS has better drive capability than PMOS, it can achieve the same current drive with a smaller size. However, the NMOS can only achieve a maximum charge equal to the maximum source charge below one V_th_ (where V_th_ is the threshold voltage of the NMOS transistor). Therefore, we use a bootstrap rectifier to raise the NMOS gate voltage one V_th_ above that of the maximum input voltage, and we allow NMOS to act like a PMOS switch to achieve the maximum potential for the general output terminal. The right half of the circuit diagram is the bootstrap circuit, where the circuit principals operate as follows. When V_A_ is high and V_B_ is low, M_N1_, M_N2_ and M_N3_ conduct, while M_P1_, M_N4_ and M_N5_ are closed. At this time, the capacitor CB is charged by V_A_. When the high and low states swap, M_N1_, M_N2_ and M_N3_ are closed, while M_P1_, M_N4_ and M_N5_ are open. The M_N5_ gate voltage will exceed the maximum input voltage such that VRectifier can reach VC2 with virtually no pressure drop, thus completely resolving the diode voltage waste produced by the bridge rectifier. Although this rectifier overcomes the problem of the bipolar voltage drop, it still poses a reverse current problem with the cross-coupled rectifier, resulting in power loss and reduced conversion efficiency.

### 3.7. Full-Wave Active Rectifier

Comparing the above architectures, we find that the bridge rectifier achieves the goal of reducing the diode voltage drop, but later structures are unable to prevent the inevitable countercurrent problem. However, charging and discharging occurs with every half wave cycle, thus increasing the power consumption above the threshold voltage of the bridge rectifier. Therefore, resolving the charge–discharge problem presents a choice between diode voltage drop and countercurrent, raising the need for an optimal method that uses at least one diode to open a conduction path because the diode itself is one of the most natural countercurrent preventions, though it still incurs a diode voltage drop. Although commercially available Schottky diodes can be used to achieve high conversion efficiency in the rectifier, their integration poses problems in terms of component size, thus limiting their applicability. A review of the architecture reveals that both diode connection and negative resistance cross-coupled connection structures act as switches, where the only difference is that the diode must conduct the threshold voltage. That is, when one terminal of the receiving coil is below the output voltage by a threshold voltage or more, the rectifier shuts off, thus limiting the maximum chargeable level of the rectifier output. The negative resistance connection is not limited by this threshold voltage. However, when the rectifier output exceeds any input, the charge stored by the rectifier output load when the other input is still low will discharge along the original charging path, resulting in reverse leakage.

### 3.8. Active Diode Rectifier

The circuit structure proposed in [9] achieves high conversion efficiency for the rectifier circuit without a voltage limit or countercurrent, which is equivalent to having a rectifier with two switch circuits (see Switch 0 and Switch 1). Assuming that VCOILa is in a higher state than VCOILb, if VCOILa is higher than Vrecout, then Switch 0 is turned on, causing the current to flow back to VCOILb through the path Switch 0 -> C_L_ -> N_2_, thus charging C_L_. However, if Vrecout is higher than VCOILa, Switch 0 may close the return path, thus completely eliminating the threshold voltage and current flow problems. Similarly, when VCOILb is higher than VCOILa, if VCOILb is higher than Vrecout, Switch 1 will turn on, causing the current to flow back to VCOILa through the path Switch 1 -> C_L_ -> N_1_. However, when Vrecout is higher than VCOILb, Switch 1 will close. This concept is referred to as an active diode rectifier architecture.

The authors in [9] proposed a special rectifier architecture, where one side of the rectifier (VCOILb versus Vrecout) is the same as the other side (VCOILa versus Vrecout). Here, only one side of the circuit structure is explained. The structure of this circuit is similar to most previously described rectifiers in that it consists of two conduction paths, one of which consists of the transistors M_P1_ and M_N1_, and these two conduction paths, respectively, pass through the positive and negative half-cycle conductors. Transistors M_7_ and M_8_ are used to keep the PMOS body at a high potential to prevent the latching effect. The M_1_–M_6_ and M_R1_–M_R6_ transistors serve as a comparator circuit. When VCOILa is higher than Vrecout, it can cause M_P1_ to completely open, and vice versa. To ensure sufficient voltage and power conversion efficiency for the entire rectifier circuit (i.e., to minimize loss), the M_R1_–M_R6_ transistors should be as large as possible to operate with large resistance. Let Vrecout be 0 V.

The current flowing through M_4_ is very small, and the gate voltage (or M_1_) can be estimated to be about Vrecout − |V_th4_| (where |V_th4_| is the absolute voltage of M_4_. Therefore, M_1_ will turn on when the gate voltage of M_1_ is less than VCOIL, which is greater than the absolute value of the limiting voltage |V_th1_| (i.e., VCOILa > Vrecout − |V_th4_| + |V_th1_|). The formula VCOILa>Vrecout − |V_th1_| shows that if |V_th4_| and |V_th1_| are equal, then M_1_ will turn on when VCOILa > Vrecout. This causes the M_6_ gate voltage to pull-up, causing M_6_ to open and M_5_ to close, while M_2_ is also closed and M_P1_ is turns on. On the other hand, M_2_ will open when Vrecout > VCOILa − |V_th3_| + |V_th2_|. When M_2_ turns on, the gate voltage of M_P1_ is pulled up by Vrecout to a level sufficient to turn off M_P1_, thus eliminating the current. Here, if M_2_ is designed with a sufficiently large current driving force, the M_P1_ gate voltage will be pulled up to close to the M_P1_ level, thus almost completely eliminating the countercurrent. However, this will result in the loss of the charge stored in the Vrecout, thus reducing conversion efficiency. Therefore, the design requires careful consideration. Despite these advantages, the conduction path of this architecture includes a diode M_N1_. Thus, when the entire charging path is activated, the charge from Vrecout to the ground will be less than VCOILa − VCOILb by one M_N1_ threshold voltage |V_thN1_|. Nonetheless, since M_1_-M_6_ and M_R1_–M_R6_ have been implemented as a comparator circuit, the countercurrent can be effectively clamped.

### 3.9. Two-Stage Active Rectifier

A two-stage active rectifier in [10] can be divided into two levels. The first level is a Negative Voltage Converter (NVC), and the second level is an active diode. In the first level, the NVC circuit output point A is connected to the active rectifier input in the second level. Point B is connected to the ground, and the NVC primarily serves to ground the input from the V_in_ positive signal through M_N1_ and M_N2_. When V_in1_ > V_in2_, M_N2_ is turned on and V_in2_ is grounded, thus M_P1_ is turned on and V_in1_ is grounded at point A. Conversely, when V_in2_ > V_in1_, M_N1_ is turned on and V_in1_ is grounded, thus M_P2_ is turned on and V_in2_ is grounded at point A. Therefore, the NVC circuit can convert the positive and negative half-cycles to a half-cycle signal that is positive compared to the GND. The benefit of this is that only terminal A requires an active diode, but the cost will be reflected in the operating frequency of the active diode. When the positive and negative half-cycles use the same diode, the comparator’s operating frequency will double. In other words, the comparator’s bandwidth and speed must be increased, while the power consumption of the active diode MPS is not reduced.

### 3.10. Highly Efficient Active Rectifier

For the above-mentioned active full-wave rectifier, the authors in [9] propose a comparator-based active diode, but the conversion efficiency of this approach is limited by the ability of the gate voltage to drive the switch. Thus, a driving buffer is needed to improve the slew rate. A similar problem is frequently found in DC-DC power conversion circuits. On the other hand, the authors in [10] use a total of six large transistors, of which four are used for half-wave conversion, and one MPBD is used as a passive diode, and MPS is the active diode. This results in reduced size efficiency. In contrast with RFID specifications, active rectifiers typically operate at a frequency of 13.56 MHz. In other words, the comparator operating frequency should be higher than this, but the diode proposed in [10] requires an operating frequency of 27.12 MHz for normal operation. In view of this, a two-stage high-frequency rectifier is unsuitable, and the authors in [11] provide a better driving force for active diodes. In this approach, the use of a comparator and driving buffer improve the delay caused by the countercurrent.

This active rectifier uses four key conduction current flow: two PMOS and two NMOS transistors, respectively called P_1,2_ and N_1,2_. In [11], the external control CTL [0:3] compensates for the improved comparator. To drive the larger P_1,2_ transistor at a high frequency of 13.56 MHz, one typically adds a buffer after the comparator as a driver. The comparator’s operating speed is limited by a time delay (TP) to determine the output response speed as the input speed changes. Due to the TP-High-to-Low (TP-HL) delay, the comparator is not in time to turn on P_1,2_ and reduce the power transmission. This delay is transmitted to the load. In addition, the TP-Low-to-High (TP-LH) delay causes the comparator to delay the closing of P_1,2_. These delays cause the PCE to fall when V_in_ < V_REC_, and the charged current is returned from the C_L_ to the secondary side coil. The TP cannot fall to zero, thus a common gate comparator (CG CMP) is used and two offset control modules (OffsetF and OffsetR) and a current shortage (CS) inverter are provided. The input of the CG comparator that injects the programmable deviation current OS-F and OS-R is dependent on the feedback status signals FB-F and FB-R of the V_out_. Thus, V_out_ time can be used to perceive rising and falling signals in advance.

With regard to the sensing timing of FB-F and FB-R, when V_in1_ − V_in2_ > V_REC_, V_out_ decreases. The CG comparator outputs the V_out_ low voltage to open P_1,2_ and causes the V_in_ current to charge to V_REC_. At this point, the FB-F signal is low, and only the OffsetF module injects current into the CG comparator. Thus, before the V_in1_ − V_in2_ voltage reaches V_REC_, V_out_ is forced to fall. When V_in1_ − V_in2_ < V_REC_, FB-R falls, followed by V_out_, opening the offset block to inject the current into the CG comparator, thus forcing V_out_ to rise when V_in1_ − V_in2_ is lower than V_REC_. However, this incurs some serious side effects. If OffsetR turns on after V_out_ begins to fall, this instantaneous negative feedback mechanism limits V_out_ from falling completely and affects V_out_ jitter. The experimental results simulating a C_L_ of 10 UF and a frequency measure of 13.56 MHz produces a maximum PCE of 84.5%. The size of each transistor and offset comparator rectifier is optimized for operation at 13.56 MHz. However, PCE decreases due to the comparator delay at higher frequencies. At lower frequencies, the PCE also drops slightly, turning off the rectifier earlier due to a fixed comparison offset.

The authors in [11] provide a better switch-driving force and suggest that the offset current can be injected to control the judgment level of the comparator to achieve an early decision by the comparator and thus reduce the countercurrent. Therefore, in recent years, more studies have focused on improving effective control of the comparator to allow the entire system to automatically compensate and nearly eliminate the countercurrent. Additionally, improving switching accuracy will improve the power conversion rate (PCE).

### 3.11. Adaptive Delay-Compensated Active Rectifier

The authors in [12] implement a mechanism for automatic compensation. The authors in [12] show the active diode circuit architecture, including a push–pull common-gate comparator, a switching delay compensation circuit, a drive circuit and a power transistor. The push–pull common-gate comparator has a high slew rate and response speed and has been increasingly used in research over the past few years. This study adopts the same method used in [11] to adjust the delay by injecting current into the comparator.

The compensation method uses the gate switch signal VGN1 to obtain a sample when the switch is turned on for the difference between the inputted Vac1 voltage and the ideal voltage. This voltage difference is used to bias the source of the input current. Switching on and off causes different delays and raises the need for using two paths to obtain independent control of the on/off offset. This design provides more accurate delay compensation for independent determination and control.

In the high-performance rectifier, the timing of turning on the active diode is important. Thus, sensing and adjusting the power transistor gate (VGN1) signal is the most basic method of correcting the delay. The delay compensation circuit is not turned on until after about 30 us, and Iac1 shows that the active diode generates a large countercurrent due to the delay, resulting in a relatively low voltage conversion rate (VCE) and power conversion efficiency (PCE).

The converted output voltage VDC is also low. When the delay compensation circuit is turned on, it tends to lock at about 100 us. The results show that the active rectifier has no inverse current, and the output voltage VDC is also higher, with a higher VCE and PCE. The PCE measurements, showing a significant increase in both the load change and input and output voltage changes after dynamic compensation is activated, indicate the importance of precision compensation.

There have recently been more works presented in the literature [13,14,15,16,17,18,19]. Despite the different design philosophies, the underlying purposes are similar. We compare all of them (Table 2) to give the readers a comprehensive idea about what achievements have been made for the applications. More perspectives on the application of MEMS to implantable microelectronics such as retinal chips can be found in [20,21].

In today’s biomedical energy-harvesting applications, as mentioned in the previous section, the power source strength may not be always stable. From the research results of reference [22], we know that one can use a time-multiplexing mechanism to perform energy harvesting and achieve maximum power point tracking (MPPT). References [23,24,25] mention different ways to improve the energy-harvesting systems.

In [23], the authors mentioned various issues and tradeoffs in design, as well as the definition of MPPT along with how to measure it. In terms of biomedical energy harvesting, to achieve the same concept of MPP, it is necessary to perform impedance matching under the premise of the target back-end load and the given limited input, to achieve the maximum rectifier power output.

In [24], the design optimization technology is especially introduced for the application of several micro-scale energy-harvesting systems. The application and circuit technology of the multiple-stage rectification and multiple-input multiple-output energy harvesting are introduced.

In [25], an emerging as well as promising technology known as a supercapacitor is discussed, along with how to achieve MPPT based on energy stored in supercapacitors through circuit technology. Although solar-powered energy harvesting is its applied object, the study still has considerable technical reference value. Although the studied points in these papers are not the same as in this review paper, they can enrich the content and depth.

Modeling, characterization, and feasibility studies of capacitive coupling for power delivery and data communication have been performed in [26,27,28,29,30,31,32]. In [32], a near-field capacitive coupling-based wireless powering scheme in the subGHz frequency range is presented, where it could safely deliver up to 100 mW of power to an implant with a peak operating efficiency of over 50%. In [33], Lee et al. proposed a voltage-boosted current-mode wireless power receiver for directly charging a low-voltage battery in implantable medical systems, where the technique is studied to charge low-voltage batteries wirelessly for supplying medical implantable systems. In [34], an inductive voltage/current mode integrated power management with seamless mode transition and energy recycling is proposed for robust inductive power delivery, based on parasitic bulk diodes of lower voltage drop.

The authors in [35,36,37,38,39,40,41,42,43] presented several novel circuit techniques to improve conversion efficiency of the rectification for MHz-range coupling. A design including both voltage- and current-mode operation covering a wide range of coupling ratios between the coils is proposed in [34]. The so-called dual-loop adaptive delay compensation, voltage mode switched offset comparator, single-stage regulating, dynamically controllable comparator, adaptive delay time control, and single-stage AC-DC converter techniques have been proposed to achieve better active rectification operating at 13.56 MHz [36,37,38,39,40,41]. In [44], an integrated resonant regulating rectifier has been presented for 144-MHz RF inputs ranging from 0.98 to 1.5 V, where rectification and regulation is combined in a single stage.

A method to transmit power and to communicate data in the reverse direction over only one pair of inductive coils is described in [45]. The authors in [46] demonstrate that robust ultrasonic power-up and data uplink are able to be realized for implantable applications. A high-efficiency ultra-low-power CMOS rectifier is presented in [47] for RF-powered wearable medical devices, where a novel self-compensated cross-coupled design is proposed and realized. In [48], a new method is proposed for compensating coupling factor variations between transmitting and receiving coils for inductive power transfer. The papers in [49,50] concern data transmission over inductive link, where state-of-the-art modulation/demodulation techniques are presented.

### 3.12. PCE Estimation Model for the Active Full-Wave Rectifier

**Figure 7:**
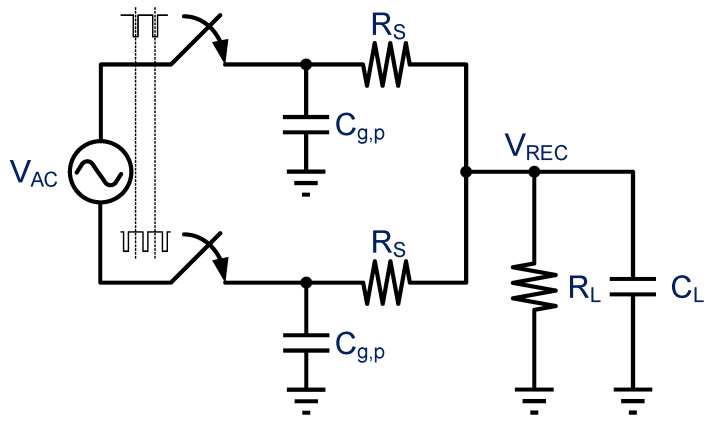
Calculation model for the active full-wave rectifier.

Figure 7 shows an active full-wave rectifier power-conversion model used to estimate the PCE. In the figure, C_g,p_ is capacitance at the active diode capacitor gate terminal, and R_S_ is output resistance when the active diode conduction is turned on. R_L_ and C_L_ are the output resistor and storage capacitor, respectively. The amount of charge is calculated using Equation (2) below, while the charge consumption is calculated using Equation (3), where I_R_S__ and I_R_L__ are, respectively, the current flowing through R_S_ and R_L_, and D is the conduction duty cycle. Note that in the full-wave rectifier, a full cycle includes a single positive and negative half-cycle, thus D must be multiplied by two.

(2) $$Q_{ch}=C_{L}\times \Delta V=\left(I_{R_{S}}-I_{R_{L}}\right)\times 2\times D\times T_{S}$$

(3) $$Q_{\mathrm{dis}}=C_{L}\times \Delta V=I_{R_{L}}\times \left(1-2D\right)\times T_{S}$$

According to the conservation of charge and discharge, we can obtain the I_R_S__ relationship as follows:

(4) $$Q_{\mathrm{ch}}=Q_{\mathrm{dis}}\to I_{R_{S}}=\frac{1}{2D}I_{R_{L}}=\frac{1}{2D}\frac{V_{\mathrm{REC}}}{R_{L}}$$

We can then obtain all the parameters needed to calculate PCE. First, the PCE formula is as follows. P_Load_ is the power consumption for output. That is, the output power is the total power consumption for the entire AC-DC conversion system.

(5) $$\mathrm{PEC}=\frac{P_{\mathrm{Load}}}{P_{\mathrm{Load}}+2\times (P_{ron,p}+P_{ron,n}+P_{swp,cg}+P_{comp})}$$

(6) $$P_{\mathrm{Load}}=\frac{{V_{\mathrm{REC}}}^{2}}{R_{L}}$$

where P_ron,p_ and P_ron,n_ are, respectively, the power consumption of the active diode and the cross-couple and are calculated using Equations (7) and (8). Because the transistor operation conduction time is in the deep triode region, the transistor parameters can be used to calculate on-resistance using Equation (9). The switching power dissipation P_swp,cg_ at the transistor gate can be calculated using Equation (10), where the remaining P_comp_ in Equation (5) is the power dissipation of the comparator and other auxiliary circuits.

(7) $$\begin{array}{cc} P_{ron,p} & ={I_{R_{S}}}^{2}\times D\times R_{on,p}\\ & ={\left(\frac{1}{2D}\frac{V_{\mathrm{REC}}}{R_{L}}\right)}^{2}\times D\times R_{on,p}\\ & =\frac{1}{4D}{\left(\frac{V_{\mathrm{REC}}}{R_{L}}\right)}^{2}\times R_{on,p} \end{array}$$

(8) $$R_{ron,n}=\frac{1}{4D}{\left(\frac{V_{\mathrm{REC}}}{R_{L}}\right)}^{2}\times R_{on,n}$$

(9) $$R_{on}=\frac{1}{\mu C_{\mathrm{ox}}\frac{W}{L}\left(V_{\mathrm{GS}}-V_{\mathrm{TH}}\right)}$$

(10) $$P_{swp,cg}=C_{g,p}\times {V_{\mathrm{REC}}}^{2}\times f_{s}$$

## 4. Conclusions and Discussion

In this paper, we mainly detail the wireless energy transfer applications involved from a circuit point of view and the existing developments in the rectification technology, where the foundation of wireless powering of energy harvesting for medical applications has also been introduced. The preceding architecture review shows that active rectifiers provide better energy conservation than passive rectifiers, but the passive architecture is not necessarily obsolete. Active diodes consume power for the comparator and compensation circuits, typically from 10s to 100s of μW, with additional switching power dissipation from hundreds of μW to several mW, confirming that given low input terminal energy (<1 mW), the active diode alone may not have sufficient power, and thus the rectifier may be unable to provide good PCE.

Therefore, an architecture should be selected based on the application scope. When the input source energy is lower than the mW level, hybrid passive rectifiers provide a practical solution, despite incurring a V_th_ pressure drop. This architecture is still capable of performing normal AC-DC energy conversion, and most digital circuits do not function properly without sufficient input power. On the other hand, higher input source energy levels offer more architecture options. In the field of biomedical applications, output power is usually below 100 mW, which means the input power must be above 100 mW, and an internal circuit power consumption of several mW is still within the acceptable range, making such applications suitable to use active diode architectures, such as a comparator.

However, when input/output power reaches several watts, the size of the active diode grows, and the delay caused by the driver exceeds the comparator’s ability to compensate. For active diodes, most high-power rectifiers use a voltage-controlled delay line (VCDL) to directly adjust the timing of active diode switching. While such circuits consume up to 10s of mW of power, this is negligible for applications with inputs/outputs measuring several watts, so we can classify the architecture by application level. Despite the fact that active rectifier structures are able to provide higher voltage conversion efficiency compared with the passive designs demonstrated in the literature, their power conversion efficiency may be compromised due to the designer’s skills.

Care must be taken to ensure that both the efficiency and hardware cost are excellent, otherwise it becomes meaningless to adopt such architectures. In general, the design of the rectifier first needs to consider the application specifications to determine the semiconductor process best suited, such as near-filed coupling, far-field coupling, or if it will be used for high-power biomedical applications (e.g., implants that require electrical stimulation) and so on.

In addition, it is necessary to consider whether the battery can be placed in the body for charging, or whether the operation of the implanted chip in the body can only be carried out by means of wireless power delivery. Moreover, the relative distance between the external platform and internal chips, such as whether it is easy to produce displacement affecting the difficulty of rectifier design, is also of primary importance. If the internal data must be sent back through the same rectifier for structure simplicity, the design will be more complicated.

Therefore, after the semiconductor process is determined for specific specifications, in addition to the impedance matching of the target load and the receiving coil, it is also necessary to pay attention to whether the implanted chip that receives power at the downstream stage has specific requirements for the power supply quality of the rectifier output. Last but not least, the system developer should also pay attention to the limitations caused by the reduced complexity of the design itself, after simplifying the rectifier circuit, to see if the desired conversion efficiency can be achieved under compromise.

Future research and development trends for the entire range of applications focus on finding ways to reduce the countercurrent and circuit power consumption in order to continuously reduce consumption to levels of several μW, allowing for low-voltage active rectifiers of high-efficiency energy harvesting.

## Figures and Tables

**Table 1:** Three major wireless charging technology standards.

	WPC	A4WP	PMA
Logo	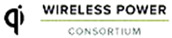	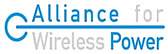	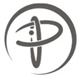
Technology	Inductive Coupling + Magnetic Resonance	Magnetic Resonance	Inductive Coupling
Main members	Philips Panasonic HTC	Samsung Qualcomm NXP	Duracell-Powermat BlackBerry NEC Starbucks
Members	214	120	71
Products	>750	0	10
Phone integrated	>80	0	0
Market	Phone/industry	Phone/tablet	Phone/tablet

**Table 2:** Comparison of recent prior articles.

Reference	[13]	[14]	[15]	[16]	[17]	[18]	[19]
Year	2016	2015	2016	2014	2016	2017	2018
Frequency	6.78 MHz	6.78 MHz	6.78 MHz	13.56 MHz	13.56 MHz	6.78 MHz	13.9 MHz
Efficiency	91.5%	84%	84%	90.1%	94%	72.6%	0.39%
Description	Full CMOS active rectifier	Full bridge rectifier	Class-E current-driven rectifier	Full wave active rectifier	Passive rectifier	Reconfigurable Rectifier	Tissue-Channel
Power	10 W	10 W	20 W	10 mW	3.2 kW	NA	NA
